# eHealth and telemedicine: Practices and beliefs among healthcare professionals and medical students at a medical university

**DOI:** 10.1371/journal.pone.0213067

**Published:** 2019-02-28

**Authors:** Anna Wernhart, Susanne Gahbauer, Daniela Haluza

**Affiliations:** 1 Medical University of Vienna, Center for Public Health, Department of Environmental Health, Vienna, Austria; 2 Medical University of Vienna, Center for Public Health, Department of Social and Preventive Medicine, Vienna, Austria; University of Helsinki, FINLAND

## Abstract

Digitalization affects almost every aspect of modern daily life including healthcare delivery. Successful adoption and sustainable integration of information technology-based eHealth and telemedicine concepts in clinical practice depend on constant evaluation of end user needs, proficiencies, and preferences. We therefore assessed how current and future healthcare professionals perceived health technology solutions and whether their perceptions differed. We conducted an online survey among a purposive sample of employees and students at the Medical University of Vienna, Austria. The structured questionnaire collected self-reported practices and beliefs in the context of eHealth and telemedicine among 905 participants (59.0% females), of which 48.4% were employees and 51.6% were students. Participants expressed moderate knowledge of eHealth and telemedicine concepts with higher levels among employees compared to students (both: p<0.05). Compared to employees, students were less convinced that online health information improves patient knowledge (p<0.001), but were more optimistic that telemedicine reduces healthcare costs (p<0.05). Participants doubted that telemedicine services would enhance the doctor-patient relationship and raised concerns regarding data security and privacy issues. Accordingly, quantitative context analysis of free text comments revealed that the four most frequently mentioned themes were related to issues concerning data privacy and security, questions of responsibility, doctor-patient interaction, and reliability of information. This study provides valuable insights into how current and future healthcare professionals differ in their perceptions regarding eHealth and telemedicine. These findings raise awareness of the need to bridge the gap between digital age groups and professional groups, especially in clinical healthcare delivery in a clocked-through, strenuous academic setting as found at a medical university.

## Introduction

The current digital revolution pushes healthcare delivery into a new age [1]. Associated structural and ideological changes as well as patient empowerment currently transform traditional, hierarchical face-to-face healthcare [2]. The term eHealth, electronic health, serves as a generic umbrella term for the use of information and communication technologies (ICT) in health-related services and processes [3,4]. eHealth has become crucial for modern healthcare systems worldwide and covers a wide variety of applications, including electronic health records, electronic medication overview, and telemedicine-related services [5,6,7]. Basically, the term telemedicine refers to the ICT-supported provision or support of health services while patients and healthcare providers are not present at the same place. In this context, ensuring secure transmission of text, sound and image-based medical data, which are per se perceived as sensitive content, are a prerequisite for medical prevention, diagnosis, treatment, and follow-up [4].

Along with rising public acceptance and distribution of consumer-oriented technologies such as smartphones, healthcare provision nowadays requires at least basic ICT with Internet access [8,9]. Broens et al. showed that technology acceptance of healthcare professionals are influenced considerably by their respective attitudes and perceptions [10]. Thus, healthcare professionals are key for national eHealth and telemedicine adoption by influencing the success of implemented ICT-based health solutions considerably [11,12,13]. Studies showed that doctors overemphasize potential barriers over benefits, with lacking financial incentives and resources, inter-operability, and regulatory frameworks on confidentiality and privacy being perceived as the main obstacles [14,15]. Ideally, stakeholders from academia, government, and industry likewise are involved at all stages of developing and implementing innovative concepts and sharing best practice examples for advancing healthcare services to overcome these barriers [16,17,18].

The Austrian healthcare sector is well equipped for using eHealth and telemedicine services. Internet access is established almost nationwide [19]. Also, eHealth literacy is likely to rise continuously with increasing IT skills of a population shifting from digital immigrants to digital natives. This concept of digital age follows Prensky`s influential definition of a younger population born digital, i.e. digital natives, and an older one that has to adapt to a rapid digitalization, i.e. digital immigrants [20,21]. However, as in other countries, Austrian citizens are barely familiar with the concepts of eHealth and telemedicine [22,23]. Moreover, many advanced services such as telemonitoring are not yet fully integrated in Austria’s standard healthcare, mostly due to a lack of funding [24,25]. As a result, numerous ambitious pilot projects aimed at improving healthcare provision are not transformed into viable business models [26]. More profound knowledge on healthcare professionals´ beliefs regarding eHealth and telemedicine as prospective consumers and end users could assist in designing useful products for everyday doctor-patient interactions and medical decision-making in a digitalized healthcare system [16].

As little evidence is available so far for the Austrian situation, we conducted a cross-sectional survey among a purposive sample of employees and students at the Medical University of Vienna. In order to capture practices and beliefs of current and future healthcare professionals roughly representing the digital age groups digital natives and digital immigrants, we strived at investigating how employees and students in a progressive as well as traditional academic surrounding perceived eHealth and telemedicine and whether their expected barriers and benefits differed.

## Methods

### Study design

The study population for this cross-sectional study was a nonrandom purposive sample of employees and students at the Medical University of Vienna. Employees mainly consisted of medical professionals who worked at the main teaching hospital of the university, i.e. the General Hospital of Vienna, Europe’s largest university hospital, which is part of the university campus [27]. The publicly funded university was founded in 1365 and is thus one of the oldest medical schools worldwide. Today, it is the largest medical education institution in German-speaking countries with about 10,000 graduate and postgraduate students. The hospital with approximately 2,200 beds includes 26 university departments, three clinical institutes, twelve theoretical medicine centers, several highly specialized laboratories, and offers top-level care for about 4,000 patients per day. Of the approximately 7,500 total faculty staff including external teaching and project employees, about 3,500 are scientific employees and about 1,000 of these are qualified research professors.

The study was approved by the ethics and data protection committees of the Medical University of Vienna and conducted according to the principles of the Declaration of Helsinki. The survey was accessible online from April 23 to May 22, 2017 via the electronic web-based survey service of Medcampus, the university`s password-protected information management system used for research and teaching administration. All employees and students affiliated with the university had single access to the survey and received an email invitation to participate. We did not offer any incentives for participation. Reminder notifications were sent by email two weeks after the initial contact. All responses were anonymous and study participation was voluntary.

### Study questionnaire

The German study questionnaire was adapted from previously published literature [22,28]. Besides system-provided data on participants`age, gender, and professional group (employee or students), the questionnaire collected self-reported information on place of living according to geographical regions (Vienna, the capital of Austria, East, and West) as well as education (primary, secondary, and tertiary). We assessed self-rated approval for familiarity with the terms eHealth and telemedicine and respective barriers and benefits, reliability of online health information, reasonability of data exchange, usefulness of data collection to monitor a chronic illness or disability, i.e. disease monitoring, as well as to increase healthy behavior, i.e. lifestyle monitoring, using 5-point Likert scale ranging from 1 = high approval to 5 = very low approval. Further, a multiple-choice question presented nine options for search terms to evaluate prevailing online health information retrieval preferences.

The questionnaire also included the open-ended question “Is there anything else you want to tell us about eHealth and telemedicine?” to collect further freely formulated comments on perceived benefits and barriers within the study population. We conducted a qualitative content analysis of these responses by assigning the narrative answers to different categories [29]. A six-step protocol guided the process aimed at connecting quantitative and qualitative research elements, while ensuring a generalization of individual cases and allowing for an empirical interpretation of the results. When reading the text for the first time, we marked text passages, where, at a first glance, research questions were addressed. When reading the text for the second time, we classified it into the category scheme, in doing so expanded the number of categories. When reading the text for the third time, we marked and took notes of particular text passages, that illustrated the process best, e.g. at which at repetition or similarity the most succinct text passage was used. We then formulated a text that illustrated this process. We further created the evaluation with text and interview passages. At the same time, we read the text for the fourth time. Finally, we marked the evaluation text for presentation, without content and interpretation attempts. Since we collected the single free text for two different professional groups, we also stratified the narrative answers by professional groups when assigning them to different categories.

### Data analysis

All statistical analyses were conducted in SPSS Statistics for Windows (Version 24.0. Armonk, NY, IBM Corp.). Two-sided level of significance was set to p<0.05. Amount of missing values was lower the 5% with random distribution, and thus tolerated without interpolation approaches, explaining deviations from the total study population, i.e. 100%. We used descriptive statistics to summarize quantitative data by reporting means, standard deviations (SD), and percentages. Mann—Whitney U tests and cChi^2^ tests evaluated differences between professional groups. We employed median splitting to dichotomize the 5-point Likert scales basically ranging from high to low approval with lower ratings indicating higher levels of agreement across all items. Thus, we yielded the variables eHealth knowledge, telemedicine knowledge, online health information reliability, reasonability health information exchange, disease monitoring, lifestyle monitoring, and approval score. The summed answers to the eleven statements on barriers and benefits items built the approval score, with a Cronbach`s alpha of 0.873 indicating a good internal consistency. We further summed up picked amount of online search terms to create the dichotomized variable online health information retrieval with lower numbers corresponding to fewer search terms, i.e. low vs. high. We assumed that a Cronbach`s alpha of 0.633 indicated an acceptable internal consistency of this scale (nine items).

Binary logistic regression analysis (enter method) assessed the association between the approval score (dichotomized dependent variable) and the independent variables. Those were socio-demographic characteristics such as professional group (employees vs. students), gender (female vs. male), education level (primary, secondary, vs. tertiary) as well as the dichotomized scores eHealth knowledge, telemedicine knowledge, monitoring, and lifestyle adaption (all: low vs. high). We reported results of the best fitting adjusted regression models according to residual plot inspection with the highest explanatory ability using odds ratios (OR), 95% confidence intervals (CI), Nagelkerke’s R^2^, predictive values, and Log Likelihood tests.

## Results

Overall, out of the 17,596 individuals contacted via the invitation mail for study participation, 905 participated in the survey (response rate: 5.1%). Of 7,407 employees entitled to the survey, 438 participated in the online survey (employee response rate: 6.0%), whereas of 10,189 students enrolled to the Medical University of Vienna, 467 participated (student response rate: 4.6%). Average age of study participants was 34.1 years (SD 12.3) and 39.7% were males. The employee sample was not only older (mean 42.6, SD 10.4 vs. mean 26.1, SD 7.7 years), but also consisted of less males than the student sample (34.7% vs. 44.3% all: p<0.001). Employees were statistically significantly more likely to live in Vienna (overall 70.3%, 77.6% vs. 63.4%) and obtain a university degree (overall 47.3%, 67.6% vs. 28.3%, all: p<0.001).

Table 1 depicts online health information retrieval strategies. Employees were less likely to search for health-specific information online in general compared to students, with the option making a doctor’s appointment being the only exception (62.3% employees vs. 43.9% students, p<0.001). In average, study subjects searched for about seven out of eleven specific options for health-related data (mean 7.1, SD 2.0, range 1–11). Employees were less likely to indicate online health information retrieval than students (mean 6.7, SD 2.1 vs. mean 7.5, SD 1.8, p<0.001). Ranking of the eleven options revealed that searching for specific diseases, symptoms, or therapeutic options (97.3%) as well as meaning of a specific medical term (97.0%) were the most common search terms overall. Finding, comparing, or assessing a healthcare service was ranked third (84.9%), whereas effects and side effects of prescription or nonprescription medicines were also very commonly searched (around 81% both).

**Table 1:** Online health information retrieval.

Have you ever searched the internet for the following health information?	Rank	Total		Employees		Students		p#
		n	%	n	%	n	%	
		905	100	438	48.4	467	51.6	
Specific diseases, symptoms, therapeutic options	1	881	97.3	421	96.1	460	98.5	0.003*
Meaning of a specific medical term	2	878	97.0	419	95.7	459	98.3	0.001**
Finding, comparing, assessing a healthcare service	3	768	84.9	360	82.2	408	87.4	0.030*
Effect of prescription or nonprescription medicines	4	735	81.2	332	75.8	403	86.3	0.001**
Side effect of prescription or nonprescription medicines	5	733	81.0	332	75.8	401	85.9	0.001**
Fitness instructions	6	606	67.0	250	57.1	356	76.2	0.001**
Vaccinations, screening programs	7	542	59.9	235	53.7	307	65.7	0.001**
Making a doctor’s appointment	8	478	52.8	273	62.3	205	43.9	0.001**
Calorie intake, nutrition diary	9	348	38.5	147	33.6	201	43.0	0.003*
Mnemonic training	10	283	31.3	107	24.4	176	37.7	0.001**
Smoking cessation, nicotine replacement therapy	11	115	12.7	48	11.0	67	14.3	0.232
Total		905	100	438	100.0	467	100	

^#^ p from chi^2^ tests (employees vs. students,

* p<0.05,

** p<0.001

Employees rated their eHealth and telemedicine knowledge higher than students (p<0.05, Table 2). Most participants were optimistic regarding reasonability of electronic health information exchange between healthcare professionals and patients, without differences between professional groups. However, employees were less inclined towards telemedicine applications for disease monitoring (p = 0.002).

**Table 2:** Respondents’ views on eHealth and telemedicine.

	High approval	Approval	Moderate approval	Low approval	Very low approval	Mean	SD^&^	p#
	n (%)	n (%)	n (%)	n (%)	n (%)			
	**How well informed do you feel about eHealth?**							
**Employees**	28 (4.6)	85 (19.5)	185 (42.2)	91 (20.8)	45 (10.3)	3.1	1.0	0.041*
**Students**	20 (4.3)	90 (19.3)	164 (35.1)	129 (27.6)	57 (12.2)	3.2	1.0	
	**How well informed do you feel about telemedicine?**							
**Employees**	28 (6.4)	83 (18.9)	158 (36.1)	106 (24.2)	60 (13.7)	3.2	1.1	0.004*
**Students**	24 (5.1)	56 (12.0)	157 (36.6)	130 (27.8)	95 (20.3)	3.5	1.1	
	**How reliable is health information from the Internet?**							
**Employees**	30 (6.8)	201 (45.9)	181 (41.3)	18 (4.1)	4 (0.9)	2.5	0.7	0.070
**Students**	30 (6.4)	172 (36.8)	228 (48.8)	27 (5.8)	4 (0.9)	2.6	0.7	
	**How reasonable is electronic health information exchange between healthcare professionals and patients?**							
**Employees**	114 (26.0)	222 (50.7)	78 (17.8)	12 (2.7)	10 (2.3)	2.0	0.9	0.067
**Students**	143 (30.6)	208 (44.5)	83 (17.8)	22 (4.7)	4 (0.9)	2.0	0.9	
	**How useful is the collection of health data or health behavior through portable sensors and smartphone apps to monitor a chronic illness or disability?**							
**Employees**	53 (12.1)	161 (36.8)	130 (29.7)	51 (11.6)	32 (7.2)	2.6	1.1	0.002*
**Students**	80 (17.1)	202 (43.3)	103 (22.1)	56 (12.2)	17 (3.6)	2.4	1.0	
	**How useful is the collection of health data through portable sensors and recommendations for a healthy lifestyle derived from them?**							
**Employees**	3.6 (8.2)	131 (29.9)	147 (36.6)	67 (15.3)	45 (10.3)	2.9	1.1	0.121
**Students**	45 (9.6)	169 (36.2)	157 (36.6)	56 (12.0)	34 (7.3)	2.7	1.0	

^**&**^ SD: Standard deviation

^#^ P values from chi^2^ tests (employees vs. students),

* p<0.05

Regarding potential barriers and benefits of telemedicine, the statement that collecting health data via telemonitoring would improve the holistic view of the patients yielded the highest approval among participants (mean 2.6, SD 1.1, Table 3). On the other hand, participants were least optimistic that data security and privacy would be guaranteed for electronically collected health data (mean 3.5, SD 1.2). As for subgroup differences, students were statistically significantly less convinced that online health information would improve patient knowledge (p<0.001) and that telemedicine would offer location-independent health services (p = 0.031). However, students were more optimistic that telemedicine would reduce healthcare costs compared to employees (p = 0.030). Ranking of potential benefits of telemedicine revealed that location-independent health services were seen as most beneficial (mean 2.0, SD 0.9), whereas the potential for enhancing the doctor-patient relationship by telemedicine services was ranked last (mean 3.3, SD 1.1).

**Table 3:** Barriers and benefits of telemedicine (range: 1 = high approval to 5 = very low approval).

Statements on barriers and benefits telemedicine	Total (n = 905)		Employees (n = 438)		Students (n = 467)		p#
	Mean	SD^&^	Mean	SD^&^	Mean	SD^&^	
Collecting health data via telemonitoring improves the holistic view of the patients.	2.6	1.1	2.6	1.1	2.5	1.1	0.143
Telemedicine improves interaction between physicians and patients.	2.9	1.1	2.8	1.1	2.9	1.1	0.167
Online health information improves patient knowledge.	3.0	1.1	2.8	1.1	3.1	1.1	0.001**
Data security and privacy are guaranteed for electronically collected health data.	3.5	1.2	3.5	1.2	3.4	1.2	0.356
Telemedicine offers location-independent health services.	2.0	0.9	2.0	0.9	2.1	1.0	0.031*
Telemedicine reduces healthcare costs.	2.7	1.1	2.8	1.1	2.6	1.0	0.030*
Telemedicine facilitates medical care.	2.7	1.1	2.8	1.1	2.7	1.1	0.184
Telemedicine reduces multiple diagnoses.	2.8	1.2	2.8	1.2	2.8	1.2	0.656
Telemedicine enhances quality of healthcare.	2.8	1.1	2.8	1.1	2.7	1.1	0.167
Telemedicine reduces healthcare administration.	2.9	1.2	2.9	1.2	2.9	1.1	0.993
Telemedicine enhances doctor-patient relationship.	3.3	1.1	3.2	1.1	3.3	1.1	0.350

^**&**^ SD: Standard deviation,

^#^ P values from Mann—Whitney U tests employees vs. students,

* p<0.05,

** p<0.001

In total, 90 (10.0%) respondents (48 employees and 42 students) added material to the free-text comments box (Table 4). Thirteen study subjects (nine employees and four students) responding “no” were not considered for analysis. Qualitative context analysis revealed that overall the greatest concerns were raised regarding data privacy and security (15.6%), inconsistent responsibility (14.4%), doctor-patient interaction (12.2%), and reliability of information (8.9%).

**Table 4:** Binary regression analysis for the approval score, stratified by professional groups.

	Total (n = 905)				Employees (n = 438)				Students (n = 467)			
	OR#	95% CI§		p	OR#	95% CI§		p	OR#	95% CI§		p
**Socio-demographic characteristics**												
**Professional group**	1.22	0.88	1.68	0.228	-				-			
**Gender**	1.00	0.73	1.36	0.998	0.77	0.47	1.24	0.279	1.07	0.71	1.64	0.736
**Education level**	0.97	0.75	1.26	0.834	0.88	0.63	1.22	0.432	1.15	0.74	1.79	0.525
**Residence**	0.91	0.73	1.14	0.422	0.81	0.54	1.21	0.300	0.95	0.72	1.24	0.688
**Dichotomized variables**^&^												
**eHealth knowledge**	0.91	0.62	1.34	0.633	1.04	0.57	1.88	0.899	0.81	0.49	1.37	0.438
**Telemedicine knowledge**	1.32	0.91	1.91	0.150	1.10	0.62	1.95	0.737	1.53	0.93	2.54	0.097
**Online health information reliability**	1.36	1.01	1.83	0.045*	1.31	0.84	2.04	0.235	1.40	0.92	2.12	0.112
**Reasonability health information exchange**	3.10	2.11	4.56	0.001**	3.78	2.10	6.78	0.001**	2.68	1.59	4.51	0.001**
**Disease monitoring**	2.98	2.15	4.13	0.001**	4.08	2.54	6.57	0.001**	2.27	1.43	3.61	0.001**
**Lifestyle monitoring**	1.23	0.83	1.82	0.304	0.95	0.54	1.65	0.849	1.75	0.98	3.12	0.061
**Online health information retrieval**	0.74	0.55	1.01	0.058	1.16	0.72	1.85	0.540	0.52	0.34	0.79	0.002*

^#^ OR: Odds ratio,

^§^ CI: Confidence interval

*p<0.05;

**p<0.001.

^&^All scores are dichotomized (high vs. low), except from online health information retrieval (low vs. high).

The single quotes in regard to data privacy and security referred to importance and relevance of data confidentiality assurance, as eHealth and telemedicine allow for medical surveillance as well as illegal abuse through third persons alike. As for subgroup differences, employees (20.8%) reported concerns about data privacy and security more than twice as often compared to students (9.5%). Data security would require a contemporary need for action such as developing ethical regulations for the data transfer.

Examples for quotes are *“It is the “safe transmission” that creates discomfort*. *My colleagues think so too*.*”* (employee), and *“Data collection requires great responsibility*. *Medical ethics should be developed equally far*!*”*(student).

The 13 answers discussing inconsistent responsibility were heterogeneous in a sense that they referred to different activities such as collecting data, using apps or gaining IT knowledge. However, they also covered national particularities such as comments on the performance of Austria’s electronic health record system. One participant compared the Austrian healthcare system to those of other countries. This category further incorporated limitations, uncertainties, and a need of action in terms of regulatory frameworks and more precise definition of responsibilities.

Examples for quotes are *“… very suitable for collecting data but not for diagnosis or therapy …”* (employee), *“Currently*, *these things are not mature in my opinion*. *Proper framework conditions are desirable*.*”* (student), and *“In the eHealth sector*, *Austria is behind Scandinavia for years*. *It should be cooperated and copied*.*”* (student).

eHealth possibilities and devices were experienced to disrupt the traditional doctor-patient interaction. Eleven participants (12.2%) mentioned different aspects or scenarios how this could look like in practice. Some mentioned that eHealth could not replace, but only nourish the face-to-face interaction between doctor and patient. Employees (12.5%) also reported problems with self-diagnosis of patients, whereas students (11.9%) also related to the need of eHealth and Artificial Intelligence for replacing doctors in the future.

Examples for quotes are *“Virtual*, *online*, *web based—everything is wonderful*, *the doctor-patient interface stays analogue”* (employee), and *“eHealth is essential that doctors are replaced by artificial intelligence*, *the insurers cheer”* (student).

Four employees (8.3%) and four students (9.5%) reported concerns regarding reliability of the information provided, be it the difficulty to distinguish valuable from useless or even misleading information or be it the need to provide evidence-based knowledge.

Examples for quotes are *“Information from the Internet is as good as the person who writes it*. *Quality control*?*”* (employee), and *“Information should be evidence based and citations should always be provided*.*”* (student).

We performed binary logistic regression analyses for the total study population as well as for employees and students separately to identify predictors for the approval score (dependent variable, Table 4). All models showed overall good performance and were a good fit to the observed data (p>0.05). Also, Nagelkerke’s R^2^ suggested that the overall, employees, and student regression models explained roughly 20.8%, 25.3%, and 20.2%, respectively, of the variance in the outcome with predictive values of 67.3%, 72.1%, and 64.9%, respectively.

The variables reasonability health information exchange (overall: OR = 3.1, 95% CI 2.1–4.6) and disease monitoring (overall: OR = 3.0, 95% CI 2.2–4.1) predicted a high approval score in all models (all: p<0.001). The factor online health information reliability (OR = 1.4, 95% CI 1.0–1.8, p = 0.045) was a respective predictor in the overall model only, as was a low online health information retrieval (OR = 0.5, 95% CI 0.3–0.8, p = 0.002) in the student model.

## Discussion

Health technologies are becoming increasingly important in the healthcare sector. eHealth and telemedicine services have the potential to improve the quality of medical care, reduce inpatient hospital stays, and reduce treatment costs [30]. In order to maximize adoption of these services, user-oriented development of advanced systems integrating knowledge on health personnel’s views as prospective consumers is necessary. One of the aims of the present study was thus to analyze whether personal experience in clinical healthcare as measured by professional status (employee and student) influences approval of eHealth and telemedicine services. We also collected comments on perceived benefits and barriers of these services in a free text item at the end of the survey questionnaire to broaden the scope of answers and provide rich data to enhance numerical result interpretation [31].

We found that students expressed lower approval for the statement that online health information improves patient knowledge compared to employees. However, they were more optimistic that telemedicine reduces healthcare costs. Noteworthy, employees and students did not differ regarding their assessment that telemedicine beneficially impacts the holistic view of patients and the doctor-patient relationship.

Perceptions on clinical benefits of telemedicine implementation concerning medical care delivery, healthcare administration, quality of healthcare, and multiple diagnoses did not differ between professional groups. The same applies to data security and privacy for electronically collected health data; both aspects were perceived as unsolved issue across professional groups.

These similarities between the professional groups are unexpected. Interestingly, they might reflect socio-cultural effects such as the powerful influence of socialization in the same medical system and exposure to similar opinion-forming mass media on a national level.

The attitudes of the medical staff are the prerequisite for the successful integration of eHealth and telemedicine in modern medical systems [32]. To increase respective knowledge and awareness among healthcare professionals, eHealth and telemedicine should be an integral part of the medical curriculum as well as of advanced training for medical staff [33]. As expected, in our sample employees were older than students (average age 43 vs. 26 years, respectively), accounting for Prensky´s classification of digital natives vs. digital immigrants [20]. As suggested by other authors, the teaching staff, which thus mostly belongs to the digital age group of digital immigrants, should adapt their teaching strategies and instruments such as virtual learning worlds and e-learning tools to meet the needs of the current generation of medical students, i.e. digital natives [34,35]. Following principles of message-framing and positive psychology, focusing on the similarities between faculty members and students might significantly enhance cooperation in medical education and training. A result would be to build up a corporate identity in the emerging field of ICT-guided health provision, teaching, and research in an academic setting, at least at faculty level.

It is to be expected that digitalization is an irreversible process worldwide, not only for private use, but especially in the healthcare sector. So, prevalences of eHealth and telemedicine use are expected to rise worldwide until reaching nearly 100% of penetration. Currently, nearly 80% of the Austrian population uses the Internet as source for health information, making online information more relevant than asking the physician [28]. Whereas in a study from Saudi Arabia, the physician was mentioned to be the most important source for health-related information (90%), with still high use of the Internet as respective information source (60%) [36].

Retrieval of health-related Internet information among patients might be perceived as double-edged sword among healthcare professionals. While an informed patient might experience empowerment and show higher therapy compliance and self-care, doctors might also feel that their clinical decision-making is negatively impacted by distorted and inappropriate health information retrieved online [37]. Concerns regarding the reliability of online health information and requirements for quality control was also among the fourth most frequently found topics in the free text comments, reflecting the participants´ negative image of Dr. Google [38]. Also, the factor reliability of online health information predicted a high approval score indicating optimistic views on health IT in the overall regression model.

To collect so far unknown empirical data for the Austrian healthcare sector, we explored prevailing search strategies among medical staff and students. In average, participants indicated to search for about 65% of the provided search term options, with students being more likely to search for health-specific information online. We identified searching for specific diseases, symptoms, or therapeutic options, and also searching for the meaning of a specific medical term as most frequently searched information. Searching the web for information on healthcare services was even more common than searching for drug effects and side effects. Degree of eHealth and telemedicine knowledge was perceived as at best moderate across professional groups, with higher ratings among employees. These findings suggest slightly better familiarity with these concepts among our study participants compared to a population-based Austrian survey showing poor self-perceived eHealth and telemedicine knowledge levels [22]. This divergence could also result from a higher confidence in their knowledge and social desirability bias among academics.

Notably, students were more inclined towards diseases management and lifestyle modification applications when compared to employees. All participants found that electronic health information exchange between healthcare professionals and patients was reasonable. Likewise, regression analysis also identified reasonability of health information exchange, in addition to disease monitoring, as an overall predictor for a high approval score in all regression models.

Primary care physicians are in principal inclined to use health technology [39]. However, especially doctors are very skeptic about technical innovations in the health sector compared to other healthcare stakeholders [16,40]. Interestingly, the free text analysis showed that the top mentioned aspects were shortcomings of eHealth rather than perceived benefits. A closer look on the single quotes revealed that the theme doctor-patient interaction was also related to a variety of potential challenges of health technologies encountered in medical practice, mostly pointing out the perceived negative impact caused by reduced traditional face-to-face contact.

Modern health technologies affect the healing relationship between practitioners and their patients through complex social processes leading to objectification, commodification, and standardization of care, as proposed by Timmermans and Almeling [41]. Especially ethical issues need to be considered when implementing eHealth and telemedicine applications [37,42]. This could also reduce skepticism among end users in the healthcare sector. Concerns regarding reliability of the respective applications, the actual achievement of the set objectives in the supply optimization, and the difficulty of eliminating the risk of error have to be publicly discussed and addressed by developers and healthcare stakeholders.

The fear of inadequate data protection negatively influences the positive assessment of health technology among doctors [13]. Digitized data could potentially be misused and passed on to insurance carriers and corporations; damage caused cannot be reversed [43]. We found that participants were skeptic whether data security and privacy would be guaranteed for electronically collected health data. Most fee text comments were assigned to data privacy and security issues, thus qualitative context analysis also identified this theme as being most relevant to the participants. This result is in line with other related publications consistently showing privacy and security concerns among various stakeholder groups such as primary care physicians and patients [39,44]. For Austrian healthcare experts participating in a Delphi survey, data security was one of the most serious obstacles for ICT use in the areas of doctor-patient interaction, health promotion as well as telemonitoring [40].

Besides security and privacy aspects, inconsistent responsibility emerged as the second most frequently mentioned theme. Due to the lack of concise Austrian eHealth strategy, private and panel doctors currently do not receive incentives for ICT-based patient contact [25]. Also, legal and ethical considerations are not well funded yet [39,42]. Structural and organizational guidelines have to clearly define adequate business models and the role of healthcare professionals including clinicians, researchers, nurses, and students to remove skepticism against health technologies in order to further enhance their adoption [25,45].

### Limitations

The findings of this study are subject to several limitations. The survey collected self-reported survey data introducing survey response bias. Furthermore, only German-speaking people affiliated with the medical university who had Internet access at home or at work and were skilled to participate in an online questionnaire took part in this survey. This sampling was intended, however, it limits generalizability of the study results to the general population. Also, our study sample included a quite large proportion of health professionals, and knowledge and aptitudes regarding eHealth and telemedicine of health professionals and non-health professionals might differ [15,46,47].

Also, given the relatively minor differences found, the simple dichotomy that students equal digital natives and employees equal digital immigrants might not mirror the real life situation. Low overall ratings in regard to searching for online information on vaccinations and screening programs could be due to the specific situation of the Austrian healthcare system, as vaccination and screening services are mainly provided by the private and panel doctor sector rather than tertiary hospitals [24].

General limitations of online surveys include uncertainty over data validity, while place- and time-independency and costs outweigh potential shortcomings of this sampling method [48]. Although the quite low participation rate limits generalizability of the study results, it was expected in a survey among healthcare workers and also comparable to similar web-based surveys via Medcampus, which are regularly used to study views among faculty members and students for evaluation and research concerns [49].

We developed a study questionnaire that could serve as a useful instrument for further assessing developments in health technologies in larger-scale national and international studies. Since eHealth and telehealth are established research interests, the qualitative content analysis was not meant to perform a hermeneutical analysis where every detail of every sentence is interpreted. It rather contributes to the existing category schema and expands it while empirically nourishing the quantitative research instrument.

## Conclusions

Digitization in everyday medical practice has gained in importance in a short time. Although the respondents of this study were employees and students of the Medical University of Vienna and therefore closer to the medical daily routine than the average population, a large part of the respondents did not feel sufficiently informed. Our findings suggest a lack of familiarity with the concept of eHealth and telemedicine. Since the students reported more experience with eHealth and telemedicine tools than the employees, the results picture the influence of digital age on eHealth and telemedicine adoption. For the successful implementation of public strategies, acceptance of eHealth and telemedicine services by consumers is crucial. The acceptance of doctors, who have an important role as opinion leaders in the population as well as the possibility to assist in designing useful new products, is essential to fully exhaust the possibilities of novel health technologies in every-day patient care.

## Supporting information

S1 File German and English version of the study questionnaire. (PDF)

S2 File Dataset. (SAV)
