# Supplementary material for: eHealth and telemedicine: Practices and beliefs among healthcare professionals and medical students at a medical university
Source: PLoS One. 2019 Feb 28;14(2):e0213067. doi: 10.1371/journal.pone.0213067 (PMC6394957; doi:10.1371/journal.pone.0213067)
Supplement: S1 File — (PDF) [file pone.0213067.s001.pdf]

Fragebogen Medcampus (Deutsch)

**Allgemeine Informationen**					
Sind Sie Angestellte/r oder StudentIn?					
Angestellte/r					
StudentIn					
Was ist ihr Geschlecht?					
Mann					
Frau					
Was ist Ihre höchste abgeschlossene Ausbildung?					
Primär (Pflichtschule, Lehre, Fachschule ohne Matura)					
Sekundär (Matura)					
Tertiär (FH, Universität)					
Wo befindet sich Ihr Hauptwohnsitz?					
Wien					
Niederösterreich					
Anderes					
Wie alt sind Sie?					
__ __ Jahre					
**Spezifische Informationen**					
	0=nein, 1=ja				
Haben Sie schon einmal online folgende gesundheitsrelevante Informationen gesucht?					
Finden, Vergleichen, Bewerten von ÄrztInnen/medizinischen Einrichtungen	0	1			
Terminvereinbarung bei ÄrztInnen/medizinischen Einrichtungen	0	1			
Bedeutung eines bestimmten medizinischen Begriffes	0	1			
Krankheiten, Symptome und Therapiemöglichkeiten	0	1			
Nikotinersatztherapie, Raucherentwöhnungstherapie	0	1			

Kalorienzähler, Ernährungstagebücher	0	1			
Impfungen, Screeningprogramme (z.B. Brustkrebsvorsorgeuntersuchung)	0	1			
Wirkung eines rezeptpflichtigen oder rezeptfreien Medikamentes	0	1			
Nebenwirkung eines rezeptpflichtigen oder rezeptfreien Medikaments	0	1			
Gedächtnistraining	0	1			
Anleitungen zu Fitnessübungen	0	1			
	1=volle Zustimmung bis 5=sehr wenig Zustimmung				
Wie gut fühlen Sie sich über das Thema eHealth informiert?	1	2	3	4	5
Wie gut fühlen Sie sich über das Thema Telemedizin informiert?	1	2	3	4	5
Wie schätzen Sie die Zuverlässigkeit von Informationen im Internet über gesundheitsrelevante Themen (z.B. Symptome, Krankheiten und Therapiemöglichkeiten)ein?	1	2	3	4	5
Wie sinnvoll finden Sie den elektronischen Austausch von gesundheitsbezogenen Informationen oder Gesundheitsdaten zwischen ÄrztInnen und PatientInnen?	1	2	3	4	5
Wie sinnvoll finden Sie eine Erfassung Ihrer Gesundheitsdaten oder Ihres Gesundheitsverhaltens durch tragbare Sensoren und Smartphone-Apps zur Überwachung einer chronischen Erkrankung oder Befindlichkeitsstörung?	1	2	3	4	5
	1	2	3	4	5
	1	2	3	4	5
Wie sinnvoll finden Sie eine Erfassung Ihrer Gesundheitsdaten durch tragbare Sensoren und die daraus abgeleiteten Empfehlungen für einen gesunden Lebensstil?	1	2	3	4	5
Wie bewerten Sie folgende Aussagen?					
Bei elektronischer Sammlung von Gesundheitsdaten ist für ausreichenden Datenschutz gesorgt.	1	2	3	4	5
Die erweiterte PatientInnenbetreuung durch die Sammlung von Gesundheitsdaten mittels Apps und Computerprogrammen verbessert die ganzheitliche Sicht auf die PatientInnen.	1	2	3	4	5
Durch Informationen aus dem Internet sind PatientInnen besser informiert.	1	2	3	4	5
Telemedizinische Anwendungen bereichern die Beziehung zwischen ÄrztInnen	1	2	3	4	5

und PatientInnen.					
Telemedizin bietet einen ortsunabhängigen Zugang zu Gesundheitsleistungen.	1	2	3	4	5
Telemedizin reduziert die Kosten der Gesundheitsversorgung.	1	2	3	4	5
Telemedizin reduziert den bürokratischen Aufwand der Gesundheitsversorgung.	1	2	3	4	5
Telemedizin verringert unnötige Mehrfachdiagnostik.	1	2	3	4	5
Telemedizin erhöht die Qualität der PatientInnenversorgung.	1	2	3	4	5
Telemedizin verbessert die Beziehung zwischen ÄrztInnen und PatientInnen.	1	2	3	4	5
Telemedizin erleichtert die ärztliche Tätigkeit.	1	2	3	4	5
Offene Frage					
Gibt es etwas, was Sie uns zu eHealth und Telemedizin noch sagen wollen? (Freitext)					

Questionnaire Medcampus (English)

**Socio-demographic information**					
Are you employee or student?					
Employee					
Student					
What is your gender?					
Male					
Female					
What is your highest education?					
Primary (compulsory school, apprenticeship, technical school without secondary school certificate)					
Sekundär (secondary school certificate)					
Tertiär (FH, Universität)					
Where do you life?					
Vienna					
Lower Austria					
Other					
How old are you?					
__ __ years					
**Specific information**					
Have you ever searched the internet for the following health information?	0 =no, 1=yes				
Finding, comparing, assessing a healthcare service	0	1			
Making a doctor’s appointment	0	1			
Meaning of a specific medical term	0	1			
Specific diseases, symptoms, therapeutic options	0	1			
Smoking cessation, nicotine replacement therapy	0	1			
Calorie intake, nutrition diary	0	1			

Vaccinations, screening programs	0	1			
Effect of prescription or nonprescription medicines	0	1			
Side effect of prescription or nonprescription medicines	0	1			
Mnemonic training	0	1			
Fitness instructions	0	1			
	1=high approval to 5=very low approval				
How well informed do you feel about eHealth?	1	2	3	4	5
How well informed do you feel about telemedicine?	1	2	3	4	5
How reliable is health information from the Internet?	1	2	3	4	5
How reasonable is electronic health information exchange between healthcare professionals and patients?	1	2	3	4	5
How useful is the collection of health data or health behavior through portable sensors and smartphone apps to monitor a chronic illness or disability?	1	2	3	4	5
How useful is the collection of health data through portable sensors and recommendations for a healthy lifestyle derived from them?	1	2	3	4	5
How do you rate the following statements?					
Data security and privacy are guaranteed for electronically collected health data.	1	2	3	4	5
Collecting health data via telemonitoring improves the holistic view of the patients.	1	2	3	4	5
Online health information improves patient knowledge.	1	2	3	4	5
Telemedicine improves interaction between physicians and patients.	1	2	3	4	5
Telemedicine offers location-independent health services.	1	2	3	4	5
Telemedicine reduces healthcare costs.	1	2	3	4	5
Telemedicine reduces healthcare administration.	1	2	3	4	5
Telemedicine reduces multiple diagnoses.	1	2	3	4	5
Telemedicine enhances quality of healthcare.	1	2	3	4	5
Telemedicine enhances doctor-patient relationship.	1	2	3	4	5
Telemedicine facilitates medical care.	1	2	3	4	5

Open question	
Is there anything else you want to tell us about eHealth and telemedicine? (Free text)	
